# Exploring the effect of antenatal depression treatment on children’s epigenetic profiles: findings from a pilot randomized controlled trial

**DOI:** 10.1186/s13148-019-0616-2

**Published:** 2019-02-04

**Authors:** Laura S. Bleker, Jeannette Milgrom, Alexandra Sexton-Oates, Tessa J. Roseboom, Alan W. Gemmill, Christopher J. Holt, Richard Saffery, Huibert Burger, Susanne R. de Rooij

**Affiliations:** 1 Department of Obstetrics and Gynecology, Amsterdam UMC, location AMC, Meibergdreef 9, Amsterdam, 1105 AZ The Netherlands; 2 Department of Clinical Epidemiology, Biostatistics and Bioinformatics, Amsterdam UMC, location AMC, Meibergdreef 9, Amsterdam, 1105 AZ The Netherlands; 3 Austin Health, Parent-Infant Research Institute, 300 Waterdale Road, Heidelberg West, VIC 3081 Australia; 4 Melbourne School of Psychological Sciences, University of Melbourne, Parkville, VIC 3010 Australia; 5 Murdoch Children’s Research Institute—Cancer and Disease Epigenetics, Royal Children’s Hospital, Flemington Road, Parkville, Melbourne, VIC 3052 Australia; 6 Department of General Practice, University of Groningen, Hanzeplein 1, 9713 GZ Groningen, The Netherlands

**Keywords:** DNA methylation, Epigenetics, Neurodevelopment, Antenatal depression, CBT, Programming

## Abstract

**Background:**

Children prenatally exposed to maternal depression more often show behavioral and emotional problems compared to unexposed children, possibly through epigenetic alterations. Current evidence is largely based on animal and observational human studies. Therefore, evidence from experimental human studies is needed. In this follow-up of a small randomized controlled trial (RCT), DNA-methylation was compared between children of women who had received cognitive behavioral therapy (CBT) for antenatal depression and children of women who had received treatment as usual (TAU). Originally, 54 women were allocated to CBT or TAU. A beneficial treatment effect was found on women’s mood symptoms.

**Findings:**

We describe DNA methylation findings in buccal swab DNA of the 3–7-year-old children (CBT(N) = 12, TAU(N) = 11), at a genome-wide level at 770,668 CpG sites and at 729 CpG sites spanning 16 a priori selected candidate genes, including the glucocorticoid receptor (*NR3C1*). We additionally explored associations with women’s baseline depression and anxiety symptoms and offspring DNA methylation, regardless of treatment. Children from the CBT group had overall lower DNA methylation compared to children from the TAU group (mean ∆β = − 0.028, 95% CI − 0.035 to − 0.022). Although 68% of the promoter-associated *NR3C1* probes were less methylated in the CBT group, with cg26464411 as top most differentially methylated CpG site (*p* = 0.038), mean DNA methylation of all *NR3C1* promoter-associated probes did not differ significantly between the CBT and TAU groups (mean ∆β = 0.002, 95%CI − 0.010 to 0.011). None of the effects survived correction for multiple testing. There were no differences in mean DNA methylation between the children born to women with more severe depression or anxiety compared to children born to women with mild symptoms of depression or anxiety at baseline (mean ∆β (depression) = 0.0008, 95% CI − 0.007 to 0.008; mean ∆β (anxiety) = 0.0002, 95% CI − 0.004 to 0.005).

**Conclusion:**

We found preliminary evidence of a possible effect of CBT during pregnancy on widespread methylation in children’s genomes and a trend toward lower methylation of a CpG site previously shown by others to be linked to depression and child maltreatment. However, none of the effects survived correction for multiple testing and larger studies are warranted.

**Trial registration:**

Trial registration of the original RCT: ACTRN12607000397415. Registered on 2 August 2007.

## Background

Many pregnant women experience clinically significant depressive symptoms before delivery, with an estimated prevalence of 7.4 to 12.8% [1]. Mounting evidence demonstrates that children prenatally exposed to maternal depression more often have a difficult temperament [2], are more prone to develop internalizing and externalizing behavioral problems [3–7], show poorer performance on cognitive tasks [8, 9], and more often develop depression and anxiety symptoms themselves in (pre)adolescence [10–12]. One mechanism by which antenatal depression might influence susceptibility for psychopathology is by epigenetic regulation of gene expression [13, 14]. Epigenetic mechanisms regulate the activity of DNA and include post-translational histone modification, micro-RNAs, and DNA methylation [15]. In contrast to the fixed genotype, the epigenome has shown to be highly variable early in development under the influence of environmental factors [16, 17].

Animal studies have provided evidence that antenatal stress alters methylation of offspring genes involved in neurodevelopment and is associated with behavioral changes. For example, exposure to chronic stress in early gestation in mice resulted in a stress-sensitive phenotype in male offspring, showing increased immobility in the tail suspension and forced swim test and heightened hypothalamic pituitary adrenal (HPA) axis responsivity, which was accompanied by increased DNA methylation and decreased gene expression of the glucocorticoid receptor in the hippocampus and amygdala [18]. Moreover, alterations in epigenetic profiles have been shown to remain stable across generations, passing on susceptibility for emotional and behavioral disorders from one generation to the next [19].

Since 2008, many human studies have investigated associations between prenatal stress exposure and offspring gene methylation, with a special focus on *NR3C1*, coding for the glucocorticoid receptor [20]. While the reported effect sizes are usually small, increased methylation status of *NR3C1* has been linked to an increased HPA axis stress-response [21]. All studies to date are, however, observational and therefore susceptible to confounding by factors that are both associated with antenatal stress and with methylation patterns, such as maternal smoking during pregnancy [22]. Experimental designs including follow-up of children are currently scarce and urgently needed to establish causality between intrauterine exposures and later life outcomes [23].

The current study investigated effects of maternal depression treatment during pregnancy on DNA methylation profiles in the children. In the Beating the Blues before Birth (BBB) study, pregnant women with a confirmed Diagnostic and Statistical Manual of Mental Disorders, 4th Edition (DSM-IV) depressive disorder were randomized to either the intervention group, consisting of eight cognitive behavioral therapy (CBT) sessions, or to a control group, consisting of treatment as usual (TAU), which comprised case-managing by a midwife or referral to a general practitioner. Beneficial treatment effects favoring the intervention were found on maternal depression and anxiety. Anxiety symptoms significantly decreased, and depressive symptoms showed a decreasing trend nearly reaching significance, in the intervention versus the control group [24].

We hypothesized that compared to the control group, the intervention would be associated with a change in DNA methylation profiles of buccal swab DNA from the children, (1) at an epigenome-wide level, (2) at 16 a priori selected candidate genes, and (3) at promoter-associated glucocorticoid receptor (*NR3C1*) probes. We additionally explored whether severity of maternal symptoms of depression and anxiety at baseline would be associated with DNA methylation profiles in the children, regardless of treatment.

## Results

### Study sample characteristics

**Fig. 1:**
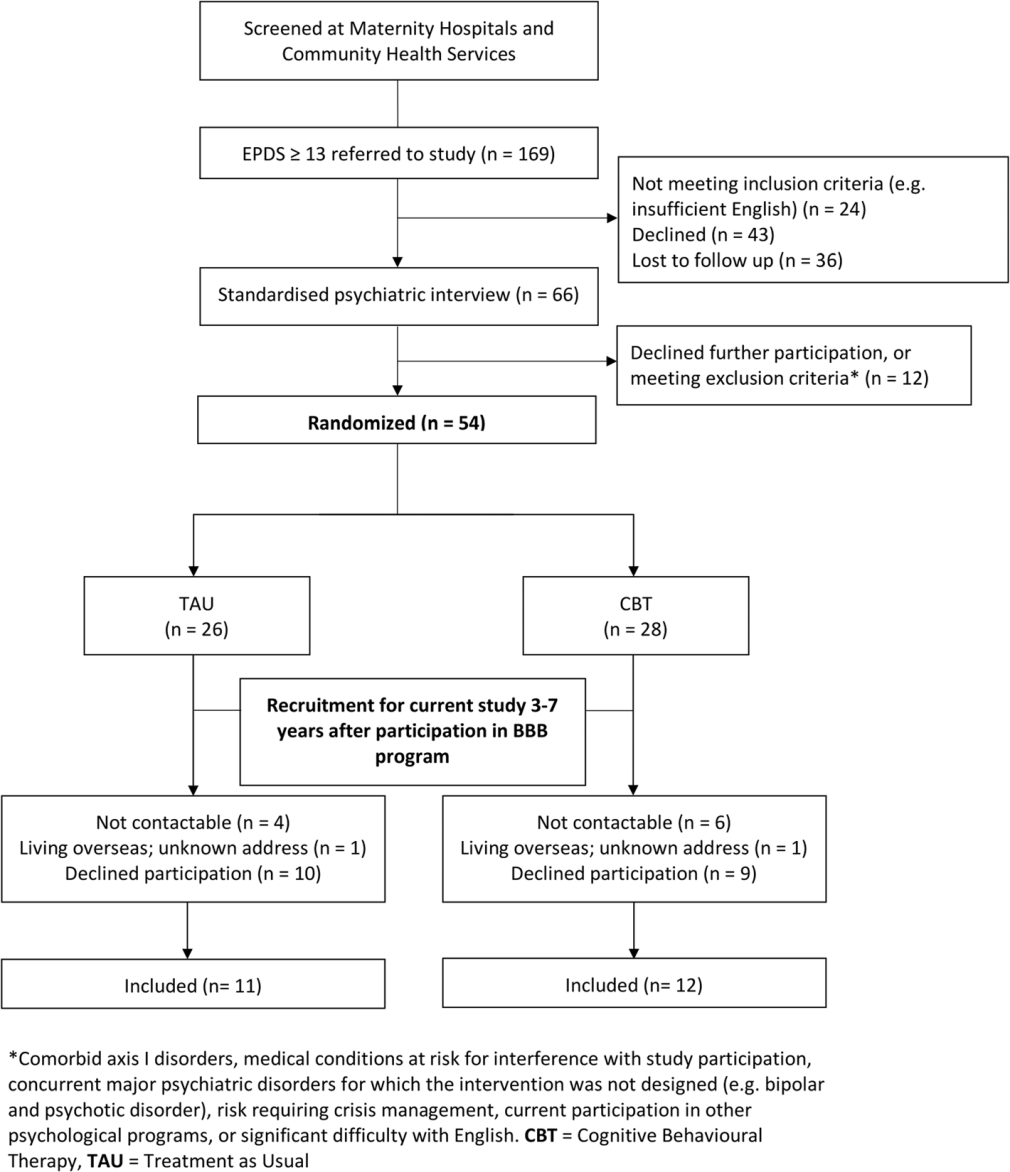
Flow diagram of participant recruitment. *Comorbid axis I disorders, medical conditions at risk for interference with study participation, concurrent major psychiatric disorders for which the intervention was not designed (e.g., bipolar and psychotic disorder), risk requiring crisis management, current participation in other psychological programs, or significant difficulty with English. *CBT* cognitive behavioral therapy, *TAU* treatment as usual

Of the original study group of 54 women, 2 women had moved overseas to unknown addresses, and 10 women could not be traced. This resulted in 42 women being invited to participate in the current study. In total, 19 women declined to participate. Reasons for declining were lack of time, a lack of interest in being involved, or not wanting their child’s DNA to be used for study purposes. This resulted in a study group of 23 women and their children who agreed to participate in the current study, 12 (42.9%) women from the intervention group and 11 (42.3%) women from the control group (flowchart; Fig. 1). Table 1 shows baseline characteristics of all women from the original study, women that did not participate, and women that did participate in the current follow-up. In the intervention and control group alike, women that responded to the current follow-up had lower Beck Depression Inventory (BDI-II) and lower Beck Anxiety Inventory (BAI) scores, less often reported using antidepressants, and were more highly educated with a higher annual income compared to non-responders at baseline. In the intervention group, participating women were more often born in Australia and married compared to women who did not participate, whereas in the control group, women were less often born in Australia and married compared to non-responders. Current demographics of the women and their children are shown in Table 2. Less women from the intervention group were currently using an antidepressant, their income was higher, and they more often drank one or more alcoholic unit per week, as compared to the control group.

**Table 1:** Baseline characteristics of all participants in a trial evaluating an antenatal cognitive behavioral therapy (CBT) versus treatment as usual (TAU), those that responded, and those that did not respond to the 5-year follow-up

	All participants		Not participating in 5-year follow-up		Participating in 5-year follow-up	
Baseline demographics	CBT (*n* = 28)	TAU (*n* = 26)	CBT (*n* = 16)	TAU (*n* = 15)	CBT (*n* = 12)	TAU (*n* = 11)
Mean (SD) BDI pre-treatment score	30.8 (9.5)	30.5 (8.9)	31.6 (9.7)	31.2 (7.8)	29.6 (9.5)	29.5 (10.4)
Mean (SD) BAI pre-treatment score	22.8 (10.0)	21.2 (10.2)	25.4 (10.1)	22.8 (12.2)	19.2 (9.0)	19.3 (7.1)
Mean (SD) BDI post-treatment score	13.0 (9.8)	17.4 (9.8)	12.9 (10.1)	17.3 (10.8)	13.0 (10.0)	17.6 (9.0)
Mean (SD) BAI post-treatment score	10.6 (7.6)	16.7 (11.8)	9.6 (5.4)	17.6 (14.3)	11.6 (9.9)	15.3 (7.1)
Mean (SD) ∆ BDI score (post-treatment − pre-treatment)	− 18.6 (10.0)	− 13.2 (12.8)	− 20.4 (12.0)	− 14.5 (10.4)	− 16.6 (7.3)	− 11.5 (16.1)
Mean (SD) ∆ BAI score (post-treatment − pre-treatment)	− 11.2 (9.4)	− 4.3 (8.3)	− 14.5 (10.1)	− 5.0 (9.8)	− 7.5 (7.2)	− 3.1 (6.0)
Mean (SD) maternal age in years	32.9 (5.9)	31.0 (5.8)	32.2 (6.5)	29.2 (5.6)	33.7 (5.7)	33.6 (5.2)
Mean (SD) gestational age in weeks	19.9 (7.7)	21.0 (6.0)	21.2 (8.0)	22.6 (6.1)	18.3 (7.2)	19.0 (5.5)
Antidepressant use (%)	7.1	22.7	14.3	26.7	–	11.1
Marital status (%)						
- Married	57.7	65.2	46.7	69.2	72.7	60.0
- De Facto	34.6	21.7	46.7	15.4	18.2	30.0
- Separated	–	8.7	–	7.7	–	10.0
- Single	7.7	4.3	6.7	7.7	9.1	–
Birth location (%)						
- Australia	73.1	82.6	66.7	84.6	81.8	80.0
- Other	26.9	17.4	33.3	15.4	18.2	20.0
Income (%)						
- Up to $ 20,000	–	4.5	–	–	–	10.0
- $ 20,001–$ 40,000	8.0	22.7	7.1	25.0	9.1	20.0
- $ 40,001–$ 60,000	20.0	13.6	28.6	16.7	9.1	10.0
- $ 60,001–$ 80,000	28.0	27.3	21.4	33.3	36.4	20.0
- > $ 80,001	32.0	31.8	28.6	25.0	36.4	40.0
- Do not wish to divulge	12.0	-	14.3	-	9.1	–
Highest level of education (%)						
- Did not finish school	3.8	12.0	6.7	21.4	–	–
- High School	7.7	24.0	13.3	21.4	–	27.3
- Certificate Level/Apprenticeship	23.1	4.0	33.3	–	9.1	9.1
- Advanced Diploma	19.2	4.0	6.7	7.1	36.4	–
- Bachelor degree	11.5	24.0	20.0	28.6	–	18.2
- Graduate diploma/certificate	19.2	16.0	6.7	7.1	36.4	27.3
- Postgraduate Degree	15.4	16.0	13.3	14.3	18.2	18.2

**Table 2:** Current characteristics of women and their children participating in a DNA methylation study

Current demographics	CBT (*n* = 12)	TAU (*n* = 11)
Mean (SD) BDI score	16.1 (13.3)	14.9 (11.2)
Mean (SD) BAI score	11.3 (8.9)	10.9 (10.2)
Mean (SD) maternal age in years	40.0 (4.9)	40.6 (4.7)
Antidepressant use, *n* (%)	2 (16.7)	6 (54.4)
Mean (SD) child age in years	5.7 (1.2)	5.9 (1.0)
Mean (SD) child birth weight in grams	3547 (332)	3520 (590)
Gender (boys) (%)	58.3	63.6
Birth location (%)		
- Australia	81.8	80.0
- Other	18.2	20.0
Marital status (%)		
- Married	66.7	54.4
- De Facto	8.3	18.2
- Separated	8.3	18.2
- Single	16.7	9.1
Highest level of education (%)		
- Did not finish school	–	–
- High School	–	27.3
- Certificate Level/Apprenticeship	8.3	9.1
- Advanced Diploma	8.3	-
- Bachelor degree	25.0	9.1
- Graduate diploma/certificate	41.7	18.2
- Postgraduate Degree	16.7	36.4
Income (%)		
- Up to $ 20,000	–	18.2
- $ 20,001–$ 40,000	8.3	18.2
- $ 40,001–$ 60,000	–	9.1
- $ 60,001–$ 80,000	8.3	9.1
- > $ 80,001	83.3	45.5
- Do not wish to divulge	–	–
Smoking^a^ (%)	8.3	9.1
Alcohol^b^ (%)	58.3	27.3

*CBT* cognitive behavioral therapy, *TAU* treatment as usual

^a,b^Defined as “currently consuming one or more alcoholic units per week or smoking one or more cigarettes per week”

### Association between genome-wide DNA methylation and allocation

Linear regression analysis was used to identify specific differentially methylated probes according to allocation. This took into account variation associated with the following covariates: birth weight, HM850 array chip position, sex and age, as identified by principal component analysis (PCA). Linear regression analysis revealed a total of 4780 differentially methylated probes at a nominal significance level (*p* < 0.01, uncorrected for multiple testing) between the intervention and the control group, showing higher DNA methylation in the control group (mean ∆β = − 0.028, 95% CI − 0.035 to − 0.022, *p* < 0.001). Adding current income as an additional covariate did not significantly alter the results (mean ∆β = − 0.026, 95% CI − 0.031 to − 0.021, *p* < 0.001). The top 100 differentially methylated probes are presented in Table 3 of the Appendix. Table 4 shows the ten most differentially methylated probes. Of the top five differentially methylated probes, three probes with annotated genes were probe cg15495292 on the *AIG1* gene (uncorrected *p* = 4.01E-06, corrected *p* = 0.999), cg05155812 on the *SUN1* gene (uncorrected *p* = 1.56E-05, corrected *p* = 0.999), and cg18818484 on the *PTCHD2* gene (uncorrected *p* = 2.20E-05, corrected *p* = 0.999). After correcting for multiple testing (corrected *p* ≤ 0.01), no probes remained significantly associated with the intervention.

**Table 3:** Top 100 differentially methylated probes according to intervention

CpG	*p*	Adjusted *p*^a^	Gene	Gene region	∆β
cg19908420	3.40E-06	0.999997557			0.049137862
cg15495292	4.01E-06	0.999997557	*AIG1*	Body	0.079710136
cg05155812	1.56E-05	0.999997557	*SUN1*	TSS1500	− 0.280713404
cg18818484	2.20E-05	0.999997557	*PTCHD2*		0.022078691
cg17622532	2.21E-05	0.999997557			0.024836631
cg14034519	2.27E-05	0.999997557	*SNX1*	Body	0.053471841
cg26436424	3.24E-05	0.999997557	*NGEF*	Body	0.033261363
cg21494953	3.48E-05	0.999997557	*C5orf23*	TSS1500	0.036133838
cg19232929	3.58E-05	0.999997557			0.054387673
cg22342380	3.86E-05	0.999997557			0.03688025
cg13719771	5.98E-05	0.999997557	*NDUFA9*	Body	0.13765872
cg10356363	6.06E-05	0.999997557	*CEBPB*	TSS1500	0.026639222
cg05205351	6.20E-05	0.999997557	*NOP56*	Body	0.05930508
cg14231326	6.23E-05	0.999997557			0.031289864
cg14358699	7.14E-05	0.999997557			0.047991502
cg06961812	8.01E-05	0.999997557	*PRODH2*	Body	0.058582642
cg16007230	8.39E-05	0.999997557	*ABCC3*	ExonBnd	0.036161879
cg25968469	8.53E-05	0.999997557	*ARHGAP22*	Body	0.056699144
cg23619591	8.80E-05	0.999997557	*C19orf81*	Body	0.057592082
cg09240747	0.000101189	0.999997557			0.067301777
cg18077049	0.000101567	0.999997557	*GLRA3*	Body	0.116790545
cg24435401	0.000110721	0.999997557	*NPAS4*	TSS1500	0.021387283
cg23274420	0.000110944	0.999997557			0.068615769
cg09223928	0.000111509	0.999997557			0.030359585
cg18666104	0.000115314	0.999997557	*CORO1C*	Body	0.058415174
cg16273469	0.000115391	0.999997557			0.036049214
cg00541777	0.000120288	0.999997557	*COLEC11*	TSS1500	0.120518141
cg06646082	0.0001208	0.999997557	*BTBD17*	TSS1500	0.0430183
cg03711840	0.000127893	0.999997557	*PLXNA1*	Body	0.043191584
cg19465002	0.000130791	0.999997557			0.033852961
cg14687471	0.000134464	0.999997557	*NBR2*	Body	0.023128809
cg27243560	0.000134814	0.999997557			0.031689225
cg05510714	0.000135017	0.999997557	*KYNU*	Body	0.153887531
cg12987887	0.000136898	0.999997557	*UPB1*	ExonBnd	− 0.01972518
cg26836955	0.000138572	0.999997557	*LONP1*	Body	0.039104166
cg26330841	0.000138665	0.999997557			0.032962344
cg16720807	0.000142967	0.999997557	*FAM176A*	5′UTR	0.042119403
cg01440210	0.000143289	0.999997557			0.030341728
cg17068417	0.000144326	0.999997557	*EEFSEC*	Body	0.030665165
cg15313810	0.000144443	0.999997557	*ST6GALNAC4*	Body	0.029787439
cg07545731	0.000147518	0.999997557	*COL22A1*	Body	0.04468122
cg14684297	0.000150469	0.999997557	*ARHGAP33*	5′UTR	0.032019831
cg10727673	0.000154265	0.999997557	*TMEM22*	TSS1500	0.089444195
cg04798314	0.000155738	0.999997557	*SMYD3*	Body	0.323390033
cg11035122	0.000160944	0.999997557	*MIR758*	TSS1500	0.055539324
cg12360330	0.000168181	0.999997557	*CENPJ*	Body	0.032193572
cg07469546	0.000172234	0.999997557			0.014405304
cg17785398	0.000172977	0.999997557	*KCNJ6*	Body	0.022656857
cg18291664	0.000173083	0.999997557	*PRKAR1B*	Body	0.040654976
cg09319487	0.000181803	0.999997557			0.033053753
cg11510586	0.000186082	0.999997557			0.107251714
cg25441526	0.000188457	0.999997557	*WDFY4*	Body	0.025251026
cg19379103	0.000188787	0.999997557	*SSBP3*	Body	0.031870653
cg19769811	0.00019183	0.999997557	*RASGRF2*	TSS1500	0.046395706
cg26221509	0.000199233	0.999997557	*SCUBE1*	Body	0.039685931
cg14700416	0.000199451	0.999997557	*SPOCK3*	5′UTR	0.049430209
cg22746421	0.000200331	0.999997557			0.02669027
cg23553242	0.000200938	0.999997557	*USP2*	Body	0.043740484
cg06617093	0.000206244	0.999997557			0.032231234
cg08670534	0.000206305	0.999997557	*COL2A1*	Body	0.032117847
cg15791944	0.000212127	0.999997557			0.055152706
cg17562896	0.000216404	0.999997557	*SV2C*	Body	0.037479302
cg02018176	0.000217297	0.999997557	*KIAA1530*	Body	0.047057842
cg11576176	0.000220243	0.999997557	*GSX2*	1stExon	0.03556139
cg09480336	0.0002295	0.999997557	*POLD1*	Body	0.03212232
cg21592262	0.000233681	0.999997557			0.06371313
cg12472342	0.000234117	0.999997557			− 0.069235248
cg18361948	0.00023564	0.999997557			0.029932491
cg00945089	0.000236572	0.999997557	*GFRA1*	Body	0.033266209
cg07442357	0.000238546	0.999997557			0.01892614
cg09193498	0.000239232	0.999997557	*SEZ6*	Body	0.042776024
cg02438610	0.000240811	0.999997557	*SUN1*	TSS1500	− 0.013139753
cg15037661	0.00024103	0.999997557	*NR1D2*	TSS1500	0.00946764
cg26264656	0.000243011	0.999997557	*SKI*	Body	0.034797294
cg24367840	0.000243465	0.999997557	*PSMD14*	Body	0.057487682
cg05289897	0.000259274	0.999997557			0.012403078
cg16419764	0.000261486	0.999997557	*CDYL*	Body	0.026043028
cg00248302	0.000266776	0.999997557	*FCRL5*	Body	0.028022889
cg24900542	0.000269678	0.999997557			0.085875055
cg15078841	0.000272298	0.999997557			0.022837528
cg12541879	0.000282436	0.999997557	*PTPRN2*	Body	0.056208383
cg01976641	0.000283246	0.999997557			0.05497368
cg17121322	0.000286514	0.999997557			0.025193249
cg17547875	0.000288231	0.999997557			0.01236688
cg18169610	0.000296554	0.999997557	*CD81*	Body	0.038708
cg04801704	0.000304651	0.999997557	*TLL2*	Body	0.025096532
cg23425290	0.000307508	0.999997557	*ABCC1*	Body	0.023343856
cg22680931	0.00030882	0.999997557	*TMEM167B*	TSS1500	0.122894387
cg01723825	0.000310423	0.999997557	*URI1*	TSS200	0.039217642
cg16261251	0.000311941	0.999997557			0.06722457
cg01400541	0.000314878	0.999997557	*C10orf128*	Body	0.042719378
cg26796807	0.000318004	0.999997557			0.04717045
cg10038145	0.000319876	0.999997557	*POR*	Body	0.045738894
cg09078103	0.000320468	0.999997557	*SNX9*	Body	0.027261168
cg08880699	0.000322485	0.999997557			0.043133838
cg03116452	0.00032398	0.999997557	*PLD3*	5′UTR	0.034421382
cg03071994	0.000324145	0.999997557	*NR4A1*	Body	0.029626215
cg21485062	0.000324634	0.999997557	*C7orf25*	Body	0.024308813
cg11504793	0.000326763	0.999997557	*NOL4L*	Body	0.025196146
cg04837576	0.00032871	0.999997557	*ADRBK2*	Body	0.030823149

∆β = mean β (treatment as usual) − mean β (cognitive behavioral therapy)

*TSS* transcription start site, *UTR* untranslated region

^a^Adjusted for multiple testing [45]

### Candidate gene-specific DNA methylation and allocation

In addition to an exploratory genome-wide analysis (above), we also tested for associations with a list of a priori chosen candidate genes. Table 5 shows the results of the unpaired Mann-Whitney-Wilcoxon tests, comparing mean DNA methylation of 16 candidate genes between the intervention and control group. No genes were significantly differentially methylated at a nominal significance level *p* < 0.01. Trends toward lower DNA methylation in the CBT group compared to the TAU group were seen in the *OXTR*, *MEST*, *MEG3*, *H19*, and *CRHR2* genes. Table 6 of the Appendix shows the probes of the candidate genes that were differentially methylated at a nominal significance level *p* < 0.01.

**Table 4:** Top 10 differentially methylated genes according to allocation

CpG	*p*	Adjusted *p*^a^	*Gene*	Gene region	Δß
cg19908420	3.40E-06	0.999998			0.049137862
cg15495292	4.01E-06	0.999998	*AIG1*	Body	0.079710136
cg05155812	1.56E-05	0.999998	*SUN1*	TSS1500	-?0.280713404
cg18818484	2.20E-05	0.999998	*PTCHD2*	Body	0.022078691
cg17622532	2.21E-05	0.999998			0.024836631
cg14034519	2.27E-05	0.999998	*SNX1*	Body	0.053471841
cg26436424	3.24E-05	0.999998	*NGEF*	Body	0.033261363
cg21494953	3.48E-05	0.999998	*C5orf23*	TSS1500	0.036133838
cg19232929	3.58E-05	0.999998			0.054387673
cg22342380	3.86E-05	0.999998			0.03688025

?ß = mean ß(TAU) – mean ß(CBT)

*CBT* cognitive behavioral therapy, *TAU* treatment as usual, *TSS* transcription start site, *UTR* untranslated region

^a^Corrected for multiple testing [46]

### The glucocorticoid receptor (*NR3C1*) gene and allocation

**Fig. 2:**
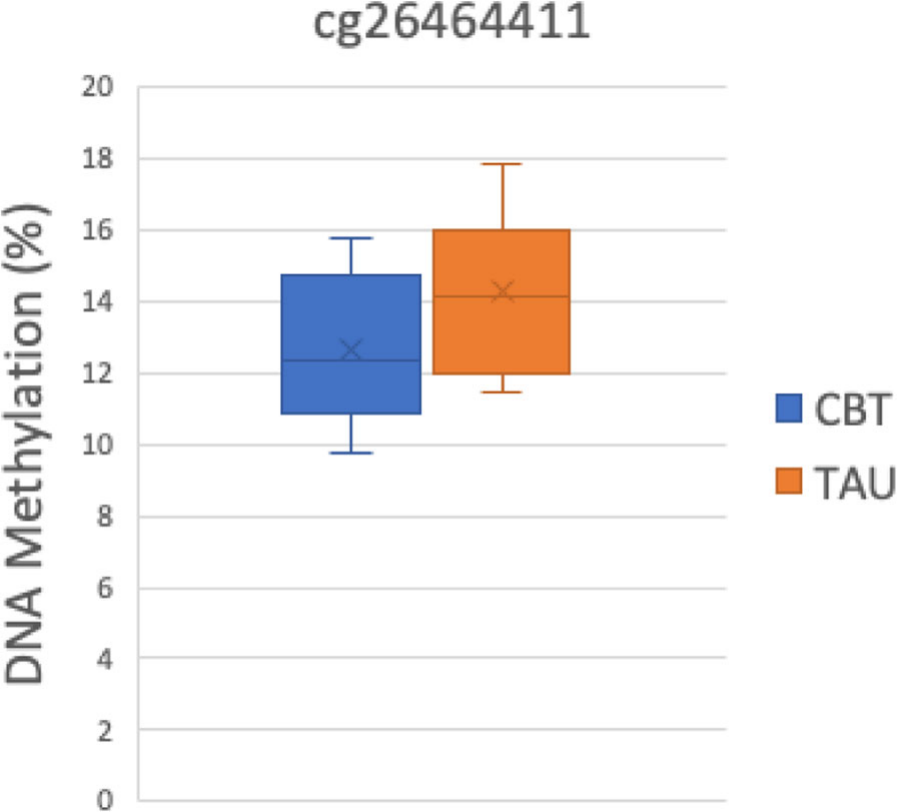
Box plot indicates methylation values (%) for children that were prenatally exposed to the intervention compared to control for the CpG site on *NR3C1* that was mostly associated with treatment exposure: cg26464411. *p* (unadjusted for multiple testing) = 0.039. *CBT* cognitive behavioral therapy, *TAU* treatment as usual

Mean DNA methylation of 34 promoter-associated *NR3C1* probes (Table 7 in Appendix) did not differ significantly between the intervention and control group (mean ∆β = 0.002, 95% CI − 0.010 to 0.011). One probe, cg26464411, showed a trend toward lower methylation in the intervention group (Table 7 in Appendix, Fig. 2).

### Association between genome-wide DNA methylation and baseline depression/anxiety

#### Depression

Linear regression analysis (adjusted for birth weight, HM850 array chip position, sex, age, and allocation) revealed a total of 3065 differentially methylated probes at a nominal significance level (*p* < 0.01) between the groups of children from the antenatally severely depressed women versus the group of children from the antenatally mildly depressed women. Mean DNA methylation values were not significantly different between children born to the severely depressed and the mildly depressed women (mean ∆β = 0.0008 95% CI − 0.007 to 0.008, *p* = 0.95). The top 100 differentially methylated probes according to depression severity at baseline are presented in Table 8 (Appendix). After correcting for multiple testing (corrected *p* ≤ 0.01), no probes remained significantly associated with maternal depression severity in pregnancy, prior to treatment.

#### Anxiety

Linear regression analysis (adjusted for birth weight, HM850 array chip position, sex, age, and allocation) revealed a total of 3292 differentially methylated probes at a nominal significance level (*p* < 0.01) between the groups of children from the antenatally severely anxious women versus the group of children from the antenatally mildly anxious women. Mean DNA methylation values were not significantly different between the children born to severely anxious and the mildly anxious women (mean ∆β = 0.0002 95% CI − 0.004 to 0.005, *p* < 0.01). The top 100 differentially methylated probes according to anxiety severity at baseline are presented in Table 9 in Appendix. After correcting for multiple testing (corrected *p* ≤ 0.01), no probes remained significantly associated with maternal anxiety severity in pregnancy, prior to treatment.

### Candidate gene-specific DNA methylation and baseline depression/anxiety

#### Depression

Table 10 (Appendix) shows the results of the unpaired Mann-Whitney-Wilcoxon tests, comparing mean DNA methylation of 16 candidate genes between the groups of children from the highly depressed and the mildly depressed women. No genes were significantly differentially methylated at a nominal significance level *p* < 0.01. Table 11 of the Appendix shows the probes of the candidate genes that were differentially methylated according to depression symptom severity at a nominal significance level *p* < 0.01.

#### Anxiety

Table 12 (Appendix) shows the results of the unpaired Mann-Whitney-Wilcoxon tests, comparing mean DNA methylation of 16 candidate genes between the groups of children from the highly anxious and the mildly anxious women. No genes were significantly differentially methylated at a nominal significance level *p* < 0.01. A trend toward higher DNA methylation was seen in the children from the highly anxious mothers compared to the children of mildly anxious mothers in the *MEST* gene. Table 11 of the Appendix shows the probes of the candidate genes that were differentially methylated according to anxiety symptom severity at a nominal significance level *p* < 0.01.

### The glucocorticoid receptor (NR3C1) gene and baseline depression/anxiety

#### Depression

Mean DNA methylation of 34 promoter-associated *NR3C1* probes (Table 13, Appendix) did not differ significantly between the groups of children from the highly depressed and the mildly depressed women (mean ∆β = 0.006, 95% CI − 0.005 to 0.020).

#### Anxiety

Mean DNA methylation of 34 promoter-associated *NR3C1* probes did not differ significantly between the groups of children from the highly anxious and the mildly anxious women (mean ∆β = 0.006, 95% CI − 0.005 to 0.020). Two probes, cg07515400 and cg22402730, showed a trend toward higher DNA methylation in the children from severely anxious mothers (Table 13, Appendix).

## Discussion

In this follow-up of one of the first randomized controlled trials on the effect of antenatal psychological depression treatment (CBT) on children’s DNA methylation patterns, we found no robust evidence of widespread methylation differences between children of women in the control or intervention group. However, at a pre-specified nominal significance level of *p* < 0.01, 4780 differentially methylated probes according to allocation pointed to an overall 2.7% lower DNA methylation level of probes in children from the intervention group. Applying a candidate approach, non-significant trends toward lower DNA methylation in the intervention group were seen in *OXTR*, *MEST*, *MEG3*, *H19*, and *CRHR2.* We did not find a significant difference in mean DNA methylation of 34 *NR3C1* promoter-associated probes between the intervention and control groups. Nevertheless, the majority of probes (68%) showed lower DNA methylation in the intervention group compared to the control group, with cg26464411 as topmost differentially methylated probe, a CpG site that has been associated with depression in earlier studies [25, 26]. Whether these trends are persistent and clinically relevant remains to be determined in future studies with larger sample size and longer follow-up.

Of the top five probes that were most differentially methylated between the intervention and the control group, three corresponded to annotated genes: cg15495292 on the *AIG1* gene, which is a gene involved in androgen regulation; cg18818484 on the *PTCHD2* gene, which is involved in neuronal proliferation and differentiation; and cg05155812 on *SUN1*, a gene that potentially plays a role in neuronal migration and cerebellar development. These findings may be relevant as the desired effect of a prenatal intervention would be to target genes that mediate the associations of prenatal stress, depression or anxiety with adverse neurodevelopmental disorders in children [27, 28]. Our results are promising, but evidently replication in larger studies is necessary.

Additionally, we revealed trends toward lower DNA methylation in children from the intervention group compared to the control group in 5 out of 16 candidate genes that have previously been associated with prenatal exposure to maternal stress, depression, or anxiety. These trends were observed in *OXTR*, the gene coding for the Oxytocin receptor; the *MEST* gene, a gene involved in metabolism; *MEG3*, a long noncoding RNA; *H19*, an imprinted gene; and *CRHR1*, a gene for corticotrophin releasing hormone receptors. We did not find a significant difference in mean DNA methylation between the intervention and control group on the promoter region of the *NR3C1* gene, coding for the glucocorticoid receptor. Nevertheless, cg26464411 showed a trend toward lower DNA methylation in the intervention group. This CpG site has been positively correlated with depressive symptoms or hypercortisolism in earlier studies [25, 26]. Although our results were not significant, the trends we have observed were in line with our expectations, based on earlier findings from observational studies showing increased methylation of *NR3C1* in newborns and young children of antenatally stressed, depressed, or anxious women [20, 29]*,* which was associated with increased stress responses [21, 30].

The women in the current study were treated at a mean of 18.6 weeks gestational age, and it may be possible that the effect of treatment on offspring DNA methylation would have been stronger if the women had been treated earlier in their pregnancies. Increased attention is currently focused on the period of early pregnancy, and even the preconception period, as an important time window for adverse environmental factors inducing prenatal programming, which has been shown in animal studies [18]. Further evidence in humans is derived from studies examining prenatal famine, in which the largest effect on offspring methylation was found after prenatal exposure to undernutrition in early pregnancy [31]. We did not test for an interaction between allocation status and gestational age on mean methylation in candidate genes because of the lack of significance in the initial analyses, but in larger future studies, exploring moderation through gestational age would be highly informative to identify treatment effects on DNA methylation during specific stages of pregnancy.

A limitation of the study was a lack of statistical power, as we were only able to include approximately half (23/54 = 43%) of the original sample in this follow-up. Nevertheless, associations between prenatal stress and methylation status of *NR3C1* have been reported in studies with a similar sample size [30, 32]. It was of interest that women who participated in the current follow-up study had lower levels of depression and anxiety at baseline compared to the participants that were lost to follow-up (Table 1). Also, they were observed to have higher incomes and were more highly educated at baseline. However, attrition bias is not likely to have occurred as this was the case in both groups [33]. Despite no formal statistical tests being conducted [34], it was evident that the difference in anxiety (BAI) scores before and after treatment between the intervention and control group was twice as high in the non-responders compared to the responders (14.5 versus 7.5), indicating that women with greater response to treatment were relatively underrepresented in the current sample. Additionally, some women in the control group also reported accessing psychological or medical treatment outside the trial [24]. This, and the lower participation of those who responded better to treatment, might have led to an underestimation of the effect of therapy on methylation profiles in the children in the current study.

Although both groups were reasonably balanced in terms of psychological and sociodemographic factors at the time of follow-up, it is still possible that other, unmeasured factors are (partly) responsible for the trends observed in the children’s epigenetic profiles according to allocation status. Because of the small sample size of our study, we chose to include only those variables that were likely to attribute mostly to the variation in DNA methylation, such as child gender, age, birth weight, and income. We did not include educational attainment, although this also appeared to be somewhat higher in the intervention group (although not statistically significant, results not shown). In addition, maternal body composition in pregnancy, pregnancy complications, and mode of delivery were not recorded in the original study files, and hence, not included in the current study. As these factors may act as mediators in the causal path from improved mood in pregnancy to better child outcomes, in future studies these variables should be included as well. Nevertheless, we did have access to the children’s birth weight, an important marker for general health of the baby, which showed to be similar between both groups. Also, we were unable to control for PC5 in the analyses, as none of the variables included in the model was associated with PC5. Nevertheless, the contribution fraction of PC5 to the variation in DNA methylation was very marginal compared to the contribution fraction of PC1, PC2, PC3, and PC4, which were associated with known variables and therefore were controlled for in our analyses. Finally, we did not adjust for cellular heterogeneity in our study. The most widely applied method is the reference-based deconvolution method originally described by Houseman et al., which permits the estimation of the proportion of various cell types within a sample based on existing reference data sets [35]. For blood, several studies have analyzed the methylation profile of the specific cell- types present in whole blood, which can serve as reference data. However, for saliva, this has not been performed systematically, but studies that have applied the Houseman deconvolution method on salivary genome wide DNA methylation data (combining reference methylomes from leucocyte subtypes and buccal epithelial cells references methylomes) have shown that saliva is less heterogenic compared to blood [36].

The impact of the postnatal environment on methylation profiles in children also cannot be ignored. Exposure to stressful life events from birth to adolescence has been associated with higher *NR3C1* methylation [37]. Although in both intervention and control group, more women were currently using antidepressant medication compared to when they were pregnant at enrollment of the original study, this was much more pronounced in the control group (relative increase of 43.3%) compared to the intervention group (relative increase of 16.7%). These observations may be consistent with a potential longer-term beneficial effect of treatment in the women, which in turn, might have positively affected child outcomes. Women from the intervention group also reported higher incomes compared to baseline, which was not the case in the control group, although including income as additional covariate did not significantly alter the results. To be able to isolate the effect of antenatal CBT on offspring DNA methylation in utero, prior to any postnatal confounding, evidence from trials that include cord blood and/or placenta samples for DNA methylation (and gene expression) are needed.

Finally, it has not yet been fully elucidated how maternal depression affects child adversity. Nevertheless, epigenetic modification of fetal genes in response to increased cortisol exposure, either directly or via a decrease in placental inactivation, has been widely accepted as a potential underlying mechanism. Although our study findings could not robustly support this hypothesis, the trends observed are in line with earlier evidence. The existing evidence is nearly exclusively based on findings from experiments in animals and observational human studies. The fact that the exploratory findings from this novel experimental study in humans are in line with the available evidence is therefore promising. It must be noted that we mostly looked at statistically significant results at an uncorrected *p*-value level. The results of our study should therefore be interpreted with caution. Although the observed effect sizes were small, with mean differences of 1–5% in methylation status, they are in line with earlier evidence [20]. Because of the lack of studies with a comparable study design, it is not yet possible to replicate our findings in a similar trial; however, plans for a larger trial are currently in progress.

## Conclusion

We found preliminary evidence of a possible effect of cognitive behavioral therapy during pregnancy on widespread methylation and a non-significant trend towards lower methylation of a specific CpG site previously linked to depressive symptoms and child maltreatment in the intervention group. However, none of the effects survived correction for multiple testing. Larger studies are now warranted.

## Methods

### Study population

For the BBB study, women aged 18 years or over, and less than 30 weeks pregnant were recruited through screening programs at the Northern Hospital and Mercy Hospital for Women, Melbourne, Australia, and via other health professionals and services in the public (e.g., obstetricians, GPs, and PaNDA; a Perinatal Anxiety and Depression helpline) and private sector (e.g., Northpark Private Hospital). The participating institutions were reached through advertisement and encouraged to refer women with suspected clinical depression. Women scoring 13 points or higher on the Edinburgh Postnatal Depression Scale (EPDS), the optimal score for detecting depression during pregnancy [38], were referred to the study for assessment by a psychologist if they consented. They were included in the study if they met DSM-IV criteria for a minor or major depressive disorder or an adjustment disorder with mixed depression and anxiety [39]. Severity of depression and anxiety symptoms was measured with the Beck Depression and Anxiety Inventories [40, 41]. Women with comorbid axis I disorders or medical conditions that were likely to interfere with study participation, risk requiring crisis management, participation in other psychological programs, or significant difficulty with English were excluded [24]. Women included in the study (*N* = 54) were randomized to receive pregnancy-specific CBT (*N* = 28) or TAU (*N* = 26). The CBT program consisted of seven individual sessions and one partner-session. TAU consisted of case-management by a midwife or a general practitioner and referral to other services of agencies as necessary. For ease of interpretation, in the results sections of this paper, the group of children of mothers from the CBT group will be referred to as the “intervention” group, and the group of children of mothers from the TAU group will be referred to as the “control” group. For participation in the current study, starting approximately 5 years after the BBB program had ended, all participants were invited through a letter. If they agreed to participate, an appointment at the Melbourne Brain Institute was planned, and informed consent was signed prior to or on the day of their visit to the clinic. If women were not able to attend the clinic, they were invited to send a buccal sample through the mail. The study was approved by the Human Research Ethics Committees of Austin Health, Melbourne, Australia.

### Data collection

A questionnaire on current sociodemographic data and current symptoms of depression and anxiety was sent to each woman’s home address. Baseline demographics, including symptoms of depression and anxiety as well as the child’s birth weight, were taken from the BBB study files. At the Melbourne Brain Centre, a cognitive assessment by means of the Wechsler Preschool and Primary Intelligence Scale (WWPSI-III) [42] was performed on the child, an MRI scan of the child’s brain was conducted, of which results are described elsewhere, and a buccal cell sample from the child was obtained by a researcher who was blinded to the allocation status of the women.

### Buccal cell samples

Buccal cells were collected using a dedicated swab (OraCollect 100, DNA Genotek Inc., Ontario, Canada). Children were instructed not to eat or drink 30 min prior to taking the swab. Women who were not able to visit the Melbourne Brain Centre were instructed how to apply the swab on their child, and asked to send the sample via mail. The swabs were stored at room temperature at the Parent-Infant Research Institute and transported to the Murdoch Children’s Research Institute (Melbourne, Australia) for DNA extraction within 2 weeks after collection.

### DNA extraction and genome-wide methylation detection

DNA extraction of all samples was performed using the NucleoBond CB20 DNA extraction kit. Purification of DNA was assessed using Nanodrop Spectrophotometry. Bisulfite conversion was performed using the EZ-96 DNA methylation kit (ZYMO Research Corporation) according to the manufacturer’s instructions. DNA methylation profiling was performed at the Australian Genome Research Facility, on bisulfite converted DNA using the Illumina Infinium Methylation EPIC BeadChip Array (HM850) (Illumina), which measures CpG methylation at > 850,000 genomic sites.

### Candidate gene approach

We extracted 729 probes spanning 16 a priori selected genes for linear regression analysis. Candidate genes were those that had previously been assessed in relation to prenatal exposure to maternal stress, depression, and/or anxiety in earlier studies [20]. Genes of interest were genes encoding brain-derived neurotrophic factor (*BDNF*; 91 probes), corticotrophin releasing hormone (*CRH*; 21 probes), corticotrophin-releasing factor-binding protein (*CRHBP*; 25 probes), corticotrophin-releasing hormone receptors 1 and 2 (*CRHR1*; 41 probes, *CRHR2*; 40 probes), FK506 binding protein (*FKBP5*; 49 probes), a long noncoding RNA (*H19*; 57 probes), hydroxysteroid 11-beta dehydrogenase 1 and 2 (*HSD11B1*; 25 probes, *HSD11B2*; 23 probes), insulin-like growth factor (*IGF2*; 15 probes), maternally expressed 3 (*MEG3*; 87 probes), mesoderm-specific transcript homolog protein (*MEST*; 63 probes), the glucocorticoid receptor (*NR3C1*; 89 probes), the mineralocorticoid receptor (*NR3C2*; 50 probes), the oxytocin receptor (*OXTR*; 22 probes), and the serotonin transporter (*SLC6A4*; 31 probes) [20]. Additionally, considering the especially strong evidence for this gene, we separately analyzed the probes of the promoter region of the glucocorticoid receptor gene (*NR3C1* promoter-associated probes; 34 probes) for differential methylation.

### Statistical analysis

DNA methylation was defined as a continuous variable varying from 0 (completely unmethylated) to 1 (completely methylated). Methylation data were processed in R using the *minfi* package. Normalization of the data was performed using the SWAN method [43]. Probes on X and Y chromosomes, probes that were associated with SNPs with a minor allele frequency > 1%, and cross-reactive probes [44] were removed from the dataset. This resulted in data for 770,668 probes available for subsequent analysis.

#### Sources of variation

**Fig. 3:**
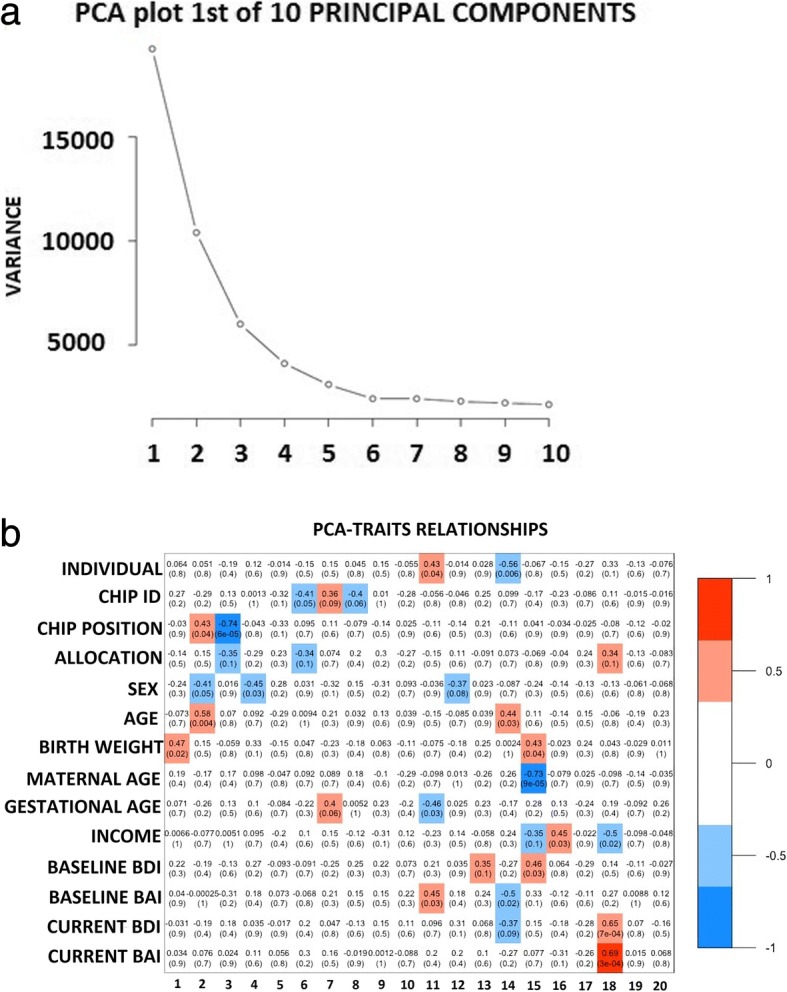
Principal component analysis results of the variation in the HM850 methylation data. Principal component analysis revealed birth weight as the major contributor to variation in the dataset with intervention status as the fifth largest contributor to variation in buccal cell DNA methylation profiles. **a** Scree plot generated with *M* values for 770,668 probes on the HM850 array. Variance is shown on the *y*-axis, principal components are shown on the *x*-axis. **b** Heatmap showing correlation coefficients, direction of correlations, and *p* values (bracketed) between principal components and various clinical parameters. Shaded boxes indicate correlations between principal components and clinical parameters (set at *p* ≤ 0.1)

**Fig. 4:**
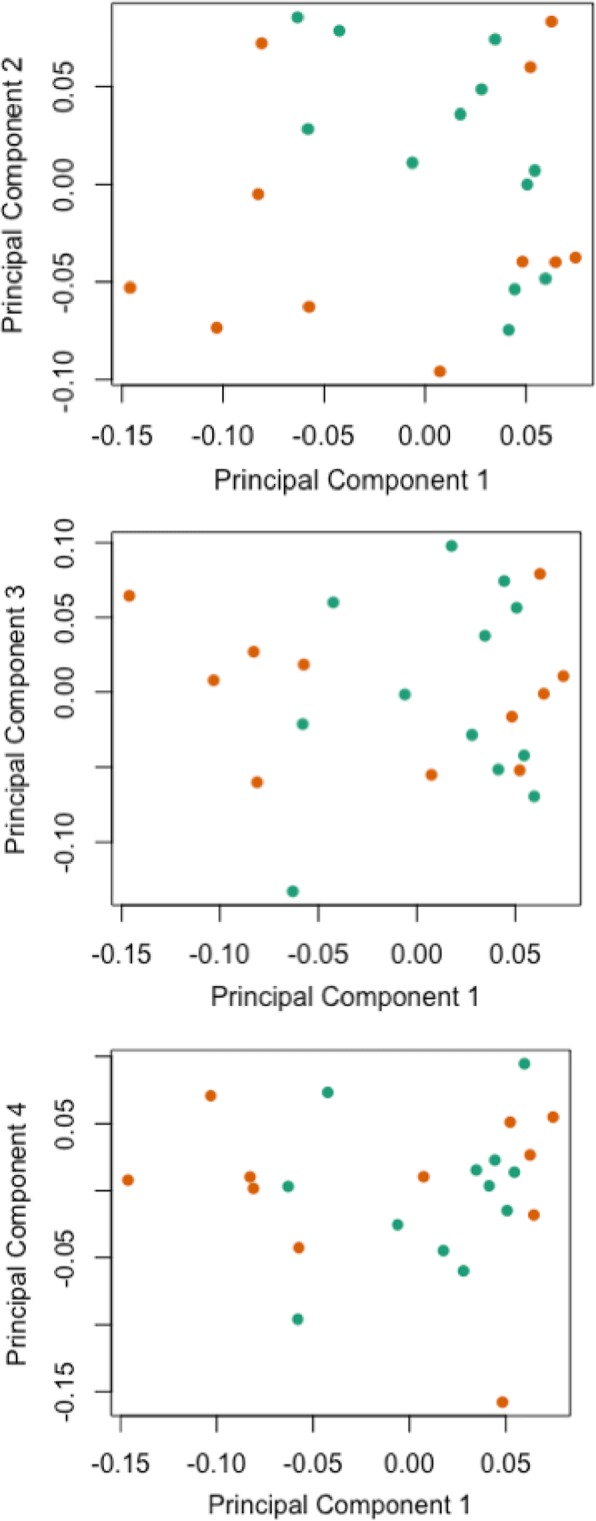
MDS plots, with samples colored according to CBT (turquoise)/TAU (orange) status, showing the relatedness of samples over the first four principal components of variation. *CBT* cognitive behavioral therapy, *TAU* treatment as usual

Main contributors to the variation in the methylation data were identified by principal component analysis (PCA). We included the following variables in the analysis to assess associations with PC’s: participant ID, chip ID, HM850 array chip position, allocation, sex, child age, birth weight, maternal age, gestational age, current income, baseline depression symptoms, baseline anxiety symptoms, current depression symptoms, and current anxiety symptoms. Results of the PCA showed that the first five principal components contributed most to the variation in the methylation data, and all variables associated with any of these PC’s were added as covariate in all analyses (Fig. 3a). The heatmap demonstrated that allocation was associated with the third principal component. Birth weight, child age, sex, and HM850 array chip position were associated with the first four principal components and they were included in the analyses as covariates. None of the variables included in our model was significantly associated with the fifth principal component, and this PC was therefore not included in our model as covariate (Fig. 3b). Unsupervised analysis by multidimensional scaling was conducted in order to examine sources of variation within the dataset. Beta values (methylation level) at all HM850 probes for all samples were used to produce multidimensional scaling (MDS) plots, with samples colored according to intervention (turquoise)/control (orange) status, showing the relatedness of samples over the first two principal components of variation (Fig. 4a). Coloring by intervention/control revealed no distinct separation by allocation. Additional MDS plots of samples over other principal components also failed to show a distinct separation between the two groups (Figs. 4b c).

#### Differential methylation according to allocation

Linear regression analysis was used to identify associations between the intervention status and epigenome-wide DNA methylation. We took into account variation associated with the covariates birth weight, HM850 array chip position, child sex and age, to account for PC1, PC2, PC3, and PC4, as identified by PCA. The Benjamini-Hochberg False-Discovery-Rate method [45] was used to correct for multiple testing. However, none of the analyses yielded significant differentially methylated probes between the intervention and control group after correcting for multiple testing. In an explorative analysis, we extracted differentially methylated probes between the intervention and control group at a nominal significance level set at *p* < 0.01, prior to correcting for multiple testing. We assessed differences in mean DNA methylation of all significant probes between the intervention and control group using an unpaired Mann-Whitney-Wilcoxon test. We additionally compared mean beta differences of 16 candidate genes, and the promoter region of the *NR3C1* gene between the intervention and control group using an unpaired Mann-Whitney-Wilcoxon test.

#### Differential methylation according to baseline depression or anxiety symptom score

As additional explorative analyses, two separate linear regression models were also used to investigate associations between baseline depression (BDI–II score) and baseline anxiety (BAI- score) with methylation profiles in the children. For ease of interpretation, the sample was divided into two groups in both analyses. The rationale behind this approach was to explore widespread methylation variation between women with severe symptoms compared to those with mild symptoms using clinically relevant cut-offs, rather than investigating the direction of correlations between increasing depression and anxiety scores on all probes separately. Baseline depression was converted to a dichotomous variable using clinically relevant Beck questionnaire cut-offs. Women with BDI-II ≥ 29 were classified as “highly depressed” (*n* = 13), whereas those with a score below 29 were classified as “mildly depressed” (*n* = 9) [46]. This procedure was repeated for baseline anxiety (BAI-score). The cut-off for clinically relevant anxiety is set at 16, and therefore we classified women with BAI ≥ 16 as “highly anxious” (*n* = 8), and women with BAI below 16 as “mildly anxious” (*n* = 14) [47]. One woman had missing data on baseline depression and anxiety and was excluded from the analysis. We took into account allocation status, birth weight, HM850 array chip position, child sex, and age as covariates, as identified by PCA. Differentially methylated probes at a nominal significance level set at *p* < 0.01, prior to correction for multiple testing, were extracted. We compared differences in mean DNA methylation in groups of children of women with high baseline symptoms and low baseline symptoms using an unpaired Mann-Whitney-Wilcoxon test, both for depression and anxiety. We additionally compared mean beta differences of 16 candidate genes, and the promoter region of the *NR3C1* gene between groups of children of women with high baseline symptoms and low baseline symptoms using an unpaired Mann-Whitney-Wilcoxon test, both for depression and anxiety.

## Appendix

**Table 5:** Differential mean methylation of candidate genes in buccal cell DNA of children after maternal antenatal CBT or TAU

Gene	Δß	95%CI	*P*
*NR3C1*	0.004	−0.004 to 0.011	0.32
*NR3C1* Promoter	0.002	−0.010 to 0.011	0.65
*SLC6A4*	0.013	−0.007 to 0.035	0.09
*OXTR*	0.008	−4.7e-05 to 1.6e-02	0.04
*NR3C2*	0.002	−0.005 to 0.009	0.6
*MEST*	0.013	0.003 to 0.024	0.02
*MEG3*	0.012	0.00004 to 0.023	0.04
*IFG2*	0.005	−0.014 to 0.028	0.65
*HSD11B1*	0.004	−0.0123 to 0.019	0.61
*HSD11B2*	0.003	−0.003 to 0.010	0.29
*H19*	0.019	0.003 to 0.041	0.03
*CRHR1*	0.013	−0.0003 to 0.027	0.06
*CRHR2*	0.019	0.002 to 0.032	0.02
*CRHRBP*	-0.003	−0.033 to 0.033	0.93
*CRH*	0.001	−0.014 to 0.015	0.98
*BDNF*	0.001	−0.005 to 0.008	0.38
*FKBP5*	0.006	−0.0003 to 0.0139	0.051

Δß = mean ß (TAU) - mean ß (CBT)

*CBT* cognitive behavioral therapy, *TAU* treatment as usual

**Table 6:** Probes in candidate gene analysis showing differential methylation according to intervention at uncorrected *p* < 0.01

CpG	*p*	Adjusted *p*^a^	Gene	Gene region	∆β
cg27338480	0.002299634	0.999997557	*MEST*	5′UTR	0.036568643
cg25579735	0.004343149	0.999997557	*NR3C1*	5′UTR	− 0.028037036
cg01913022	0.0064351	0.999997557	*CRHR2*	TSS1500	0.068307524
cg03366382	0.006909299	0.999997557	*INS-IGF2*	TSS1500	0.044997291
cg03128167	0.009155461	0.999997557	*IGF2*	Body	0.017691809

∆β = mean β (treatment as usual) − mean β (cognitive behavioral therapy)

*TSS* transcription start site, *UTR* untranslated region

^a^Adjusted for multiple testing [45]

**Table 7:** Differential methylation according to intervention (promoter-associated *NR3C1* probes)

CpG	*p*	adjusted *P*^1^	∆β
cg26464411	0.038765207	0.999997557	0.016954389
cg07515400	0.080810513	0.999997557	− 0.006695682
cg10847032	0.097881389	0.999997557	0.002994888
cg06952416	0.1418427	0.999997557	0.022027436
cg06968181	0.220252023	0.999997557	0.007404024
cg18019515	0.226633505	0.999997557	0.002112324
cg04111177	0.239037451	0.999997557	− 0.002860936
cg18068240	0.254658402	0.999997557	0.002064659
cg21209684	0.270282959	0.999997557	0.002460768
cg19135245	0.272388772	0.999997557	0.004258499
cg07733851	0.279542254	0.999997557	0.02357243
cg15910486	0.292918216	0.999997557	0.004537478
cg01967637	0.338536262	0.999997557	0.003919932
cg17860381	0.357836419	0.999997557	0.000876506
cg18849621	0.379245855	0.999997557	0.002552033
cg21702128	0.406504887	0.999997557	− 0.001070247
cg13764763	0.454791344	0.999997557	0.015622476
cg00629244	0.503885658	0.999997557	− 0.00246556
cg14939152	0.504120134	0.999997557	0.000577132
cg27122725	0.529860939	0.999997557	0.006029979
cg14558428	0.531421634	0.999997557	0.001417758
cg08818984	0.551707805	0.999997557	− 0.030134797
cg24026230	0.564518425	0.999997557	0.002507375
cg03906910	0.630630252	0.999997557	− 0.02119966
cg13648501	0.652981749	0.999997557	0.001717513
cg16335926	0.740313284	0.999997557	− 0.001532178
cg26720913	0.743323678	0.999997557	− 0.017368038
cg17342132	0.818325933	0.999997557	0.011875955
cg18718518	0.88056981	0.999997557	0.004555236
cg22402730	0.908119964	0.999997557	− 0.000126521
cg15645634	0.908177372	0.999997557	− 0.001196809
cg23776787	0.933952752	0.999997557	− 0.00580295
cg11152298	0.951420262	0.999997557	0.000520728
cg18998365	0.961116448	0.999997557	0.001743816

∆β = mean β (treatment as usual) − mean β (cognitive behavioral therapy)

*TSS* transcription start site, *UTR* untranslated region

^a^Adjusted for multiple testing [45]

**Table 8:** Top 100 differentially methylated probes according to baseline depression (BDI-II)

CpG	*p*	Adjusted *p*^a^	Gene	Gene region	∆β
cg01656717	5.43E-05	0.985858571	*WWP2*	Body	0.020713379
cg06022376	5.62E-05	0.985858571	*CACTIN*	Body	0.031934062
cg01120173	5.91E-05	0.985858571	*ZNF232*	5′UTR	− 0.032894902
cg24732447	8.42E-05	0.985858571	*OSTM1*	TSS1500	− 0.040891939
cg17402103	9.76E-05	0.985858571			0.044389084
cg10276665	0.000102293	0.985858571	*PHF20*	5′UTR	− 0.053135525
cg23119960	0.000108933	0.985858571	*TCF12*	TSS1500	0.019411015
cg07639472	0.000110211	0.985858571	*GABARAP*	TSS200	0.009595275
cg14522236	0.000112046	0.985858571			− 0.049713856
cg16561657	0.000150143	0.985858571			− 0.055819652
cg21014120	0.00015174	0.985858571	*ICA1L*	TSS200	− 0.006901363
cg02965870	0.000158156	0.985858571	*NEDD4*	1stExon	− 0.005222348
cg19817882	0.000171789	0.985858571	*LEFTY1*	Body	0.034762224
cg02644616	0.000173319	0.985858571			− 0.00792669
cg00369151	0.000179443	0.985858571	*PIP4K2A*	Body	− 0.036713932
cg24954665	0.000194603	0.985858571			− 0.018476691
cg08217452	0.000200085	0.985858571			0.062010845
cg22796353	0.000209579	0.985858571			− 0.06798597
cg05636467	0.000213261	0.985858571	*EBF3*	Body	0.058972943
cg01870580	0.000213772	0.985858571	*SGCD*	Body	− 0.033578993
cg04167481	0.000227738	0.985858571	*LRRC6*	Body	− 0.02281918
cg07010552	0.000233	0.985858571	*CHRNB1*	Body	0.03251269
cg09877950	0.000238965	0.985858571	*SLC4A10*	Body	− 0.050949676
cg08548444	0.000241925	0.985858571			0.058594659
cg22870344	0.000242958	0.985858571	*ATP5B*	TSS200	0.040824173
cg16692066	0.000251174	0.985858571	*FNDC7*	Body	− 0.027886693
cg03781315	0.00025551	0.985858571	*AHCY*	Body	− 0.020798236
cg18303019	0.000261834	0.985858571	*TXNRD1*	TSS1500	− 0.03100706
cg07381391	0.000267381	0.985858571			0.203102371
cg17115402	0.000269335	0.985858571	*CDR2L*	Body	− 0.020519014
cg23788051	0.000272662	0.985858571			0.034154924
cg15234197	0.000277725	0.985858571			0.09308691
cg22521539	0.000282937	0.985858571			0.049648454
cg25157095	0.000284638	0.985858571	*RIPK4*	Body	0.029861946
cg25464078	0.000290016	0.985858571	*PPTC7*	Body	0.041450584
cg24667213	0.000295285	0.985858571			0.021353983
cg03716908	0.00029717	0.985858571			0.036164552
cg11747082	0.000319919	0.985858571	*GPR33*	TSS1500	− 0.043309753
cg08446512	0.000321548	0.985858571	*MIR548Q*	Body	− 0.057652312
cg10239816	0.000321981	0.985858571	*GOT1*	TSS200	0.010999285
cg24632014	0.000329696	0.985858571	*LOC100189589*	Body	0.033737209
cg14255237	0.000331265	0.985858571	*SARDH*	Body	0.068885565
cg01874640	0.000341932	0.985858571	*HGD*	ExonBnd	− 0.027385445
cg12308055	0.000342843	0.985858571	*VAC14*	Body	0.025670901
cg13747435	0.000353254	0.985858571	*AK1*	Body	0.02153699
cg26287679	0.000353404	0.985858571	*MYBL1*	Body	− 0.036514738
cg27305222	0.000359452	0.985858571			− 0.040634637
cg09694986	0.000364186	0.985858571	*SNTB1*	Body	− 0.041411009
cg04928577	0.000370129	0.985858571			− 0.069549039
cg02059927	0.000376777	0.985858571			0.045567447
cg19553615	0.000379462	0.985858571	*CRTC3*	Body	0.021785594
cg06214427	0.000382521	0.985858571	*MYO1A*	Body	− 0.027829513
cg14609960	0.000388595	0.985858571	*PITRM1*	Body	− 0.03071115
cg07814876	0.000392304	0.985858571	*GGPS1*	5′UTR	0.02176791
cg03656020	0.000394532	0.985858571	*VGF*	3′UTR	0.02323939
cg16977720	0.000414373	0.985858571	*TRABD2A*	Body	− 0.015144172
cg11173076	0.00041489	0.985858571	*ART1*	TSS200	0.051579054
cg11407226	0.000427414	0.985858571			0.052957159
cg24676514	0.000428063	0.985858571			0.007114356
cg24353217	0.000430568	0.985858571	*MYL2*	Body	0.048161701
cg13022689	0.000438434	0.985858571			− 0.014053374
cg08013270	0.000452709	0.985858571	*EMX1*	Body	0.009802214
cg10486455	0.000457554	0.985858571	*WDR46*	Body	− 0.071572335
cg08824610	0.000457605	0.985858571	*SCN3B*	Body	0.032374425
cg23934072	0.00046317	0.985858571	*KIF21B*	3′UTR	0.072005944
cg08882432	0.000492053	0.985858571	*CCDC171*	Body	− 0.06825542
cg19075081	0.000509177	0.985858571	*MTSS1L*	Body	0.034977378
cg14940449	0.000513204	0.985858571	*HGS*	TSS200	− 0.004493762
cg27644292	0.000535008	0.985858571	*SNRPN*	5′UTR	− 0.043063624
cg13277044	0.000537047	0.985858571			− 0.028669058
cg10313065	0.000547596	0.985858571			0.027390264
cg27483342	0.000549745	0.985858571			− 0.035483152
cg00167525	0.00054993	0.985858571			− 0.044404069
cg02624701	0.000556261	0.985858571	*SLC17A7*	Body	− 0.023735747
cg24488506	0.000559886	0.985858571	*FOSL1*	1stExon	− 0.005249818
cg10894284	0.000567688	0.985858571	*SPATS2*	Body	− 0.05283773
cg00045787	0.0005679	0.985858571	*SNTB2*	Body	0.021702408
cg22379574	0.000572536	0.985858571	*TPT1*	TSS200	0.002542434
cg09381162	0.000579437	0.985858571	*ANXA13*	Body	− 0.038107869
cg10562399	0.000581216	0.985858571	*SNRPG*	Body	0.049683592
cg17422878	0.000584164	0.985858571			− 0.01955572
cg16460816	0.000592284	0.985858571	*IFT140*	Body	0.016906513
cg22647874	0.000594316	0.985858571	*FAM192A*	5′UTR	− 0.01781755
cg04157647	0.000594803	0.985858571	*CD27-AS1*	Body	− 0.068075731
cg14436051	0.000595366	0.985858571	*PRR26*	Body	− 0.018081196
cg11629443	0.000598589	0.985858571	*TRIM27*	1stExon	0.005616034
cg03163982	0.00059979	0.985858571			− 0.008328044
cg11475558	0.000600783	0.985858571	*TNS1*	Body	0.028042851
cg18014277	0.000608293	0.985858571	*APBB1IP*	3′UTR	− 0.016579214
cg02597373	0.000619621	0.985858571	*UNC13D*	Body	0.05993223
cg23123838	0.000622213	0.985858571	*MTA1*	TSS200	0.023497892
cg03278573	0.000627109	0.985858571	*DAP*	Body	− 0.064990789
cg15674937	0.000643134	0.985858571			0.073468304
cg01126532	0.000643521	0.985858571			− 0.081499283
cg04736676	0.000662804	0.985858571	*MCM3AP*	TSS1500	0.012334518
cg11240430	0.000664374	0.985858571	*ANKRD16*	5′UTR	0.017463959
cg00154646	0.000664385	0.985858571			− 0.022644413
cg06434997	0.000669569	0.985858571	*FBXO5*	5′UTR	0.023162548
cg02030350	0.00067758	0.985858571		Body	− 0.030538262
cg13357903	0.000693814	0.985858571	*MIA3*	TSS1500	0.012988536

∆β = mean β (severely depressed) − mean β (mildly depressed)

*TSS* transcription start site, *UTR* untranslated region

^a^Adjusted for multiple testing [46]

**Table 9:** Top 100 differentially methylated probes according to baseline anxiety (BAI)

CpG	*p*	Adjusted *p*^a^	Gene	Gene region	∆β
cg06513375	1.01E-06	0.77590274	*ZNF251*	Body	− 0.106421132
cg19573881	5.11E-06	0.998778059			0.083994741
cg00117018	1.40E-05	0.998778059	*ZNF251*	Body	− 0.13038673
cg11602361	3.18E-05	0.998778059	*FYN*	5′UTR	− 0.045924181
cg21641920	3.80E-05	0.998778059	*RBM33*	Body	− 0.056092107
cg13511253	4.12E-05	0.998778059	*MAPK4*	5′UTR	− 0.06921116
cg11674381	4.68E-05	0.998778059			− 0.030888002
cg00115113	5.01E-05	0.998778059	*LINC00483*	Body	0.027732238
cg21918548	5.84E-05	0.998778059	*ZNF251*	Body	− 0.100935223
cg01519784	5.87E-05	0.998778059			− 0.025857817
cg07081372	6.58E-05	0.998778059	*TMX1*	Body	0.020743268
cg26293081	7.19E-05	0.998778059	*TNS3*	Body	0.039738087
cg06626791	7.25E-05	0.998778059	*CCNE2*	5′UTR	0.012276869
cg04788249	7.26E-05	0.998778059	*ATG7*	5′UTR	0.003609404
cg08049441	7.76E-05	0.998778059	*RPL32P3*	Body	− 0.024015531
cg10731606	8.45E-05	0.998778059	*AGBL3*	TSS200	0.031982023
cg02335517	0.000117192	0.998778059	*IL6*	Body	− 0.013920705
cg12379948	0.00011944	0.998778059	*WNT3*	TSS1500	0.007283815
cg13242754	0.000127218	0.998778059	*C14orf101*	Body	− 0.015166989
cg06245967	0.000130491	0.998778059	*BANP*	5′UTR	− 0.029761211
cg21643916	0.000138817	0.998778059	*PRKAR1B*	Body	− 0.013338272
cg22500132	0.000147833	0.998778059	*MUC1*	TSS200	0.00823408
cg24555816	0.000150316	0.998778059			0.058918432
cg02893361	0.000160529	0.998778059	*PIAS1*	Body	− 0.030954487
cg12906188	0.000164316	0.998778059	*RGS4*	Body	0.008487123
cg05524951	0.000170319	0.998778059			− 0.012679223
cg14122980	0.000170584	0.998778059	*PTPRD*	5′UTR	− 0.023320139
cg13449967	0.000178787	0.998778059	*ATG2A*	Body	0.029533776
cg17231980	0.000185655	0.998778059			− 0.013659095
cg04657000	0.000189668	0.998778059	*FYN*	5′UTR	− 0.012205559
cg18612255	0.000205249	0.998778059			0.012801625
cg22063222	0.000229138	0.998778059			− 0.010538791
cg23760165	0.000231842	0.998778059	*FADS2*	TSS1500	0.00669263
cg24531534	0.000237063	0.998778059	*LOXL2*	Body	0.102423109
cg15745507	0.000240352	0.998778059			0.039058624
cg05731717	0.000243608	0.998778059			− 0.038853941
cg16888838	0.000245704	0.998778059	*KIAA1549*	3′UTR	− 0.021553937
cg17190403	0.000249731	0.998778059	*C6orf211*	Body	0.029783554
cg18298090	0.000274478	0.998778059	*ETV2*	TSS1500	− 0.035320986
cg11341317	0.000285303	0.998778059			− 0.032941327
cg15264808	0.000285946	0.998778059	*CENPN*	5′UTR	0.012161477
cg21025551	0.000290064	0.998778059	*ADRBK2*	TSS200	0.008238703
cg15872329	0.000304128	0.998778059	*BLOC1S2*	Body	0.010747948
cg26594377	0.000311806	0.998778059	*EFCAB11*	5′UTR	0.007818559
cg27191554	0.000311819	0.998778059	*NOTCH3*	Body	0.016376385
cg25899954	0.000314492	0.998778059			− 0.015053877
cg09602803	0.000326585	0.998778059			0.052616473
cg23462514	0.000333695	0.998778059	*RNF212*	TSS200	− 0.085842379
cg18193440	0.000336288	0.998778059	*TAF1L*	1stExon	0.071586528
cg09398891	0.000343573	0.998778059			− 0.017821675
cg08949296	0.000351898	0.998778059	*JPH1*	1stExon	0.008914397
cg21943599	0.000355323	0.998778059	*C1orf125*	TSS1500	− 0.012630326
cg04322378	0.000356074	0.998778059	*LINC01258*	TSS200	0.036276539
cg13921204	0.000358982	0.998778059	*SEC61A2*	TSS200	0.004130793
cg07346187	0.000360053	0.998778059	*ZC3H12D*	Body	0.008900888
cg11832804	0.000361177	0.998778059	*TERT*	Body	− 0.006660909
cg04899629	0.00036328	0.998778059	*LOR2C3*	TSS1500	− 0.067707778
cg01985858	0.000364399	0.998778059	*OBFC2B*	TSS1500	0.012234912
cg03851648	0.000366413	0.998778059	*PHC2*	Body	− 0.103038845
cg11102724	0.000382353	0.998778059			0.200746665
cg18570658	0.000387535	0.998778059	*COL4A2*	Body	− 0.06295432
cg24942330	0.000389195	0.998778059	*ASAH1*	TSS1500	0.005198328
cg07571142	0.00039639	0.998778059	*C10orf99*	3′UTR	− 0.022383608
cg14405643	0.000402335	0.998778059	*IER5L*	3′UTR	0.026828829
cg13147522	0.000402551	0.998778059	*SAPS3*	TSS200	0.011988911
cg15417944	0.000405638	0.998778059	*RBM44*	5′UTR	− 0.03261725
cg00616952	0.000409576	0.998778059	*SIPA1L3*	Body	− 0.019003771
cg23166923	0.000410512	0.998778059	*PMPCA*	1stExon	0.008004775
cg13297582	0.000411378	0.998778059	*LDLRAD4*	5′UTR	− 0.092481987
cg00962271	0.000413861	0.998778059			− 0.042309368
cg11640106	0.000416865	0.998778059	*LOC101929194*	Body	− 0.016168103
cg06981781	0.000418137	0.998778059	*EGF*	Body	− 0.011108508
cg24146773	0.000418853	0.998778059	*SH3BGR*	1stExon	− 0.083744695
cg23579746	0.000438092	0.998778059	*FCRLB*	TSS1500	− 0.026251069
cg09819772	0.000438692	0.998778059			− 0.019080858
cg06630983	0.000440009	0.998778059	*PPM1F*	Body	− 0.013620731
cg09207053	0.000444686	0.998778059	*PCDHGA11*	TSS200	0.021510517
cg11833983	0.000447858	0.998778059	*KANSL2*	Body	− 0.020712784
cg05675803	0.000455891	0.998778059	*C6orf52*	Body	0.006716337
cg03265692	0.000455941	0.998778059	*ATAD1*	TSS1500	0.010548663
cg11463903	0.000458655	0.998778059	*ING5*	TSS1500	0.015381351
cg03211481	0.00046527	0.998778059	*DNAJC1*	Body	− 0.022278955
cg17714799	0.000472182	0.998778059	*CASP6*	TSS1500	0.018907097
cg20034712	0.000482406	0.998778059	*ZNF836*	TSS1500	− 0.060359087
cg11554391	0.000485943	0.998778059	*AHRR*	Body	0.014764295
cg06166863	0.000490293	0.998778059	*PNN*	TSS200	0.007284371
cg26321013	0.000491445	0.998778059	*WIPF2*	1stExon	0.018566869
cg16261619	0.000495054	0.998778059	*ZPBP*	TSS200	− 0.049720147
cg06871884	0.000495095	0.998778059	*LINC00963*	Body	0.008107368
cg16333300	0.000496236	0.998778059	*TECTA*	Body	− 0.023279226
cg21848211	0.000497682	0.998778059			− 0.019228316
cg16287252	0.00050262	0.998778059	*GLT1D1*	Body	− 0.059937553
cg15568778	0.000504593	0.998778059			− 0.009418856
cg15247039	0.000514355	0.998778059			− 0.026521804
cg04800443	0.000518233	0.998778059			0.034208928
cg12937337	0.000519654	0.998778059	*PTEN*	5′UTR	− 0.020668502
cg05308125	0.000534128	0.998778059			− 0.017025401
cg13267264	0.000538758	0.998778059	*PRDM14*	TSS200	0.023761421
cg06610641	0.000538794	0.998778059	*ZNF527*	TSS1500	0.019166156
cg16642284	0.00053992	0.998778059	*FOXI2*	TSS200	0.019871733

∆β = mean β (severely anxious) − mean β (mildly anxious)

*TSS* transcription start site, *UTR* untranslated region

^a^Adjusted for multiple testing [46]

**Table 10:** Differential methylation of candidate genes according to baseline depression (BDI-II)

Gene	∆β	95% CI	*p*
*NR3C1*	0.002	− 0.006 to 0.011	0.647
*NR3C1* Promoter	0.006	− 0.005 to 0.020	0.2093
*SLC6A4*	0.004	− 0.022 to 0.028	0.647
*OXTR*	0.003	− 0.009 to 0.010	0.5123
*NR3C2*	− 0.002	− 0.012 to 0.007	0.647
*MEST*	0.009	− 0.002 to 0.018	0.1264
*MEG3*	0.007	− 0.008 to 0.0120	0.2921
*IFG2*	− 0.006	− 0.035 to 0.018	0.6005
*HSD11B1*	− 0.002	− 0.022 to 0.017	0.7938
*HSD11B2*	0.004	− 0.005 to 0.009	0.2093
*H19*	0.016	− 0.011 to 0.039	0.2624
*CRHR1*	0.008	− 0.016 to 0.0221	0.3575
*CRHR2*	0.005	− 0.014 to 0.0246	0.647
*CRHRBP*	0.011	− 0.0262 to 0.0372	0.5556
*CRH*	− 0.006	− 0.017 to 0.009	0.3237
*BDNF*	0.003	− 0.001 to 0.010	0.1641
*FKBP5*	0.003	− 0.023 to 0.031	0.7414

∆β = mean β (severely depressed) − mean β (mildly depressed)

**Table 11:** Probes in candidate gene analysis showing differential methylation according to baseline depression (BDI-II) at uncorrected *p* < 0.01

CpG	*p*	Adjusted *p*^a^	Gene	Gene region	∆β
cg17578833	0.002812934	0.985858571	*CRH*	TSS1500	− 0.055448723
cg04137760	0.00427371	0.985858571	*FKBP5*	5′UTR	− 0.028541521
cg08077673	0.007257559	0.985858571	*MEST*	5′UTR	0.008859633
cg07583420	0.00759847	0.985858571	*IGF2*	Body	0.00580552
cg13167664	0.009158888	0.985858571	*IGF2*	Body	0.003859675

∆β = mean β (severely depressed) − mean β (mildly depressed)

*TSS* transcription start site, *UTR* untranslated region

^a^Adjusted for multiple testing [45]

**Table 12:** Differential methylation of candidate genes according to baseline anxiety (BAI)

Gene	∆β	95% CI	*p*
*NR3C1*	− 0.006	− 0.013 to 0.004	0.2382
*NR3C1* Promoter	0.008	− 0.001 to 0.019	0.0817
*SLC6A4*	0.005	− 0.022 to 0.028	0.5699
*OXTR*	− 0.004	− 0.015 to 0.005	0.3301
*NR3C2*	0.004	− 0.007 to 0.011	0.4411
*MEST*	0.013	0.001 to 0.023	0.01965
*MEG3*	0.012	− 0.0005 to 0.025	0.06983
*IFG2*	− 0.004	− 0.028 to 0.026	0.7135
*HSD11B1*	0.009	− 0.008 to 0.027	0.2667
*HSD11B2*	0.005	− 0.003 to 0.012	0.11
*H19*	0.014	− 0.009 to 0.039	0.2382
*CRHR1*	0.003	− 0.015 to 0.021	0.6163
*CRHR2*	0.001	− 0.018 to 0.024	0.9734
*CRHRBP*	0.016	− 0.021 to 0.045	0.4411
*CRH*	− 0.007	− 0.019 to 0.006	0.402
*BDNF*	0.007	− 0.0007 to 0.011	0.0817
*FKBP5*	0.005	− 0.003 to 0.014	0.145

∆β = mean β (severely anxious) − mean β (mildly anxious)

**Table 13:** Differential methylation according to baseline anxiety (BAI) (promoter-associated *NR3C1* probes)

CpG	*p*	Adjusted *p*^a^	Gene region	∆ß
cg07515400	0.019543236	0.998778059	TSS1500	0.008757408
cg22402730	0.034941814	0.998778059	TSS1500	0.007846464
cg18068240	0.074595651	0.998778059	5'UTR	0.003513867
cg00629244	0.075603387	0.998778059	TSS200	0.005153991
cg21209684	0.095644074	0.998778059	5'UTR	0.005819462
cg17860381	0.172486506	0.998778059	5'UTR	−0.007857672
cg18849621	0.181055644	0.998778059	TSS1500	0.007722928
cg26720913	0.22868997	0.998778059	1stExon	0.071542284
cg16335926	0.238092713	0.998778059	TSS1500	0.002021547
cg24026230	0.24577813	0.998778059	5'UTR	0.005938353
cg18019515	0.245786808	0.998778059	TSS200	0.001549976
cg23776787	0.295148027	0.998778059	1stExon	0.055157644
cg11152298	0.296050346	0.998778059	TSS200	0.003074345
cg17342132	0.318902496	0.998778059	Body	−0.021285299
cg27122725	0.347102162	0.998778059	5'UTR	0.035263092
cg10847032	0.355174482	0.998778059	TSS1500	−0.000186142
cg21702128	0.358888726	0.998778059	TSS1500	0.003153478
cg26464411	0.373131972	0.998778059	TSS1500	0.008722328
cg18998365	0.436272234	0.998778059	5'UTR	0.005641782
cg06968181	0.486353571	0.998778059	TSS1500	0.004702526
cg03906910	0.524841281	0.998778059	1stExon	0.051231481
cg14939152	0.572528468	0.998778059	5'UTR	−0.003292981
cg04111177	0.59537643	0.998778059	5'UTR	0.002463121
cg06952416	0.665139695	0.998778059	5'UTR	0.036482196
cg08818984	0.673915225	0.998778059	1stExon	0.043838345
cg13648501	0.723256995	0.998778059	5'UTR	0.008987005
cg19135245	0.779311238	0.998778059	TSS1500	0.001611098
cg07733851	0.816930585	0.998778059	5'UTR	0.029161123
cg15645634	0.864608392	0.998778059	5'UTR	−0.002973062
cg01967637	0.911624523	0.999388148	5'UTR	−0.004461401
cg14558428	0.913633924	0.999388148	5'UTR	0.000461939
cg18718518	0.937744615	0.999400864	TSS1500	0.023943315
cg13764763	0.939306554	0.999410992	TSS1500	0.012472219
cg15910486	0.975291537	0.999632341	5'UTR	−0.003768688

?ß = mean ß (severely anxious) - mean ß (mildly anxious)

*TSS* transcription start site, *UTR* untranslated region

^a^Adjusted for multiple testing [45]

**Table 14:** Probes in candidate gene analysis showing differential methylation according to baseline anxiety (BAI) at uncorrected *p*?<?0.01

CpG	*p*	Adjusted *p*^a^	Gene	Gene region	∆ß
cg26880525	0.00670039	0.998778059	*HSD11B1*	5'UTR	−0.07833178
cg07704699	0.007191379	0.998778059	*BDNF*	Body	0.026796044
cg13670288	0.007464434	0.998778059	*IGF2*	Body	−0.003490477
cg23273257	0.009092701	0.998778059	*NR3C1*	3'UTR	−0.014724328

?ß = mean ß (severely anxious) - mean ß (mildly anxious)

*TSS* transcription start site, *UTR* untranslated region

^a^Adjusted for multiple testing [45]

**Table 15:** Differential methylation according to baseline depression (BDI-II) (promoter-associated *NR3C1* probes)

CpG	*p*	Adjusted *p*^a^	Gene region	∆β
cg22402730	0.09232524	0.985858571	TSS1500	0.007914958
cg07515400	0.14983598	0.985858571	TSS1500	0.00381786
cg18849621	0.155838866	0.985858571	TSS1500	0.011301146
cg27122725	0.191928791	0.985858571	5′UTR	0.04731291
cg19135245	0.244476459	0.985858571	TSS1500	0.005449225
cg01967637	0.254982491	0.985858571	5'UTR	− 0.002502373
cg21702128	0.310632555	0.985858571	TSS1500	0.003068137
cg06968181	0.341577022	0.985858571	TSS1500	0.015388067
cg26464411	0.354871977	0.985858571	TSS1500	0.018949214
cg14558428	0.355258299	0.985858571	5′UTR	0.000239683
cg00629244	0.377208819	0.985858571	TSS200	− 0.003861647
cg08818984	0.399423704	0.985858571	1stExon	0.000741573
cg23776787	0.447963073	0.985858571	1stExon	0.016867225
cg13648501	0.469320108	0.985858571	5′UTR	0.016976965
cg03906910	0.497810362	0.985858571	1stExon	0.010141716
cg18068240	0.512386308	0.985858571	5′UTR	0.00411793
cg21209684	0.572146062	0.985858571	5'UTR	0.00264155
cg16335926	0.591526062	0.985858571	TSS1500	0.003497235
cg04111177	0.611636013	0.985858571	5′UTR	0.000989473
cg13764763	0.65429048	0.985858571	TSS1500	0.008619545
cg14939152	0.795018613	0.988065713	5′UTR	0.001196214
cg26720913	0.798815567	0.988185518	1stExon	0.029770303
cg18998365	0.856200047	0.991299612	5′UTR	0.016860787
cg07733851	0.871021038	0.992356236	5′UTR	0.022062569
cg18718518	0.873514581	0.992390392	TSS1500	0.015863129
cg06952416	0.87457104	0.992509068	5′UTR	0.033670117
cg17860381	0.875979533	0.992611179	5′UTR	0.000759159
cg18019515	0.916972995	0.995093105	TSS200	0.000584247
cg11152298	0.925587204	0.995541393	TSS200	1.94E-05
cg17342132	0.936691926	0.996297376	Body	− 0.021376556
cg15645634	0.948521192	0.997183288	5′UTR	− 0.001498486
cg24026230	0.951640736	0.997379305	5'UTR	0.002161506
cg10847032	0.979982104	0.998978654	TSS1500	0.004273011
cg15910486	0.985159251	0.999341633	5′UTR	0.003121248

∆β = mean β (severely depressed) − mean β (mildly depressed)

*TSS* transcription start site, *UTR* untranslated region

^a^Adjusted for multiple testing [45]

## Abbreviations

BAI: Beck Anxiety Inventory
BBB: Beating the Blues before Birth
BDI-II: Beck Depression Inventory-II
CBT: Cognitive behavioral therapy
DSM-IV: Diagnostic and Statistical Manual of Mental Disorders, 4th Edition
MDS: Multidimensional scaling
PCA: Principal component analysis
RCT: Randomized controlled trial
TAU: Treatment as usual

## References

[1] Bennett HA, Einarson A, Taddio A (2004). Prevalence of depression during pregnancy: systematic review. Obstet Gynecol.

[2] Huizink AC, Robles de Medina PG, Mulder EJH (2003). Stress during pregnancy is associated with developmental outcome in infancy. J Child Psychol Psychiatry.

[3] Hay DF, Pawlby S, Waters CS (2010). Mothers’ antenatal depression and their children’s antisocial outcomes. Child Dev.

[4] Gerardin P, Wendland J, Bodeau N (2011). Depression during pregnancy. J Clin Psychiatry.

[5] Korhonen M, Luoma I, Salmelin R (2012). A longitudinal study of maternal prenatal, postnatal and concurrent depressive symptoms and adolescent well-being. J Affect Disord.

[6] Leis JA, Heron J, Stuart EA (2014). Associations between maternal mental health and child emotional and behavioral problems: does prenatal mental health matter?. J Abnorm Child Psychol.

[7] Van Batenburg-Eddes T, Brion MJ, Henrichs J (2013). Parental depressive and anxiety symptoms during pregnancy and attention problems in children: a cross-cohort consistency study. J Child Psychol Psychiatry.

[8] Barker ED, Jaffee SR, Uher R (2011). The contribution of prenatal and postnatal maternal anxiety and depression to child maladjustment. Depress Anxiety.

[9] Koutra K, Chatzi L, Bagkeris M (2013). Antenatal and postnatal maternal mental health as determinants of infant neurodevelopment at 18 months of age in a mother–child cohort (Rhea Study) in Crete, Greece. Soc Psychiatry Psychiatr Epidemiol.

[10] Davis EP, Sandman CA (2012). Prenatal psychobiological predictors of anxiety risk in preadolescent children. Psychoneuroendocrinology.

[11] Pawlby S, Hay DF, Sharp D (2009). Antenatal depression predicts depression in adolescent offspring: prospective longitudinal community-based study. J Affect Disord.

[12] Pearson RM, Evans J, Kounali D (2013). Maternal depression during pregnancy and the postnatal period. JAMA Psychiatry.

[13] Saffery R (2014). Epigenetic change as the major mediator of fetal programming in humans: are we there yet?. Ann Nutr Metab.

[14] Novakovic B, Saffery R (2013). The importance of the intrauterine environment in shaping the human neonatal epigenome. Epigenomics.

[15] Murphy TM, Mill J, Dick K (2014). Epigenetics in health and disease: heralding the EWAS era. Lancet (London, England).

[16] Schroeder JW, Conneely KN, Cubells JF (2011). Neonatal DNA methylation patterns associate with gestational age. Epigenetics.

[17] Gudsnuk K, Champagne FA (2012). Epigenetic influence of stress and the social environment. ILAR J.

[18] Mueller BR, Bale TL (2008). Sex-specific programming of offspring emotionality after stress early in pregnancy. J Neurosci.

[19] Drake AJ (2004). Intergenerational consequences of fetal programming by in utero exposure to glucocorticoids in rats. AJP Regul Integr Comp Physiol.

[20] Ryan J, Mansell T, Fransquet P (2017). Does maternal mental well-being in pregnancy impact the early human epigenome?. Epigenomics.

[21] Oberlander TF, Weinberg J, Papsdorf M (2008). Prenatal exposure to maternal depression, neonatal methylation of human glucocorticoid receptor gene (NR3C1) and infant cortisol stress responses. Epigenetics.

[22] Joubert BR, Felix JF, Yousefi P (2016). DNA methylation in newborns and maternal smoking in pregnancy: genome-wide consortium meta-analysis. Am J Hum Genet.

[23] Van Lieshout RJ, Krzeczkowski JE (2016). Just DO (HaD) It! Testing the clinical potential of the DOHaD hypothesis to prevent mental disorders using experimental study designs. J Dev Orig Health Dis.

[24] Milgrom J, Holt C, Holt CJ (2015). Feasibility study and pilot randomised trial of an antenatal depression treatment with infant follow-up. Arch Womens Ment Health.

[25] Glad CAM, Andersson-Assarsson JC, Berglund P, et al. Reduced DNA methylation and psychopathology following endogenous hypercortisolism—a genome-wide study. Nat Publ Gr. 2017; 10.1038/srep44445. Epub ahead of print 10.1038/srep44445 PMC5353706 28300138

[26] Radtke K, Schauer M, Gunter H, et al. Epigenetic modifications of the glucocorticoid receptor gene are associated with the vulnerability to psychopathology in childhood maltreatment. Transl Psychiatry. 2015;5 10.1038/tp.2015.63. Epub ahead of print 10.1038/tp.2015.63 PMC4471294 26080088

[27] Deave T, Heron J, Evans J (2008). The impact of maternal depression in pregnancy on early child development. BJOG An Int J Obstet Gynaecol.

[28] Field T, Diego M, Hernandez-Reif M (2006). Prenatal depression effects on the fetus and newborn: a review. Infant Behav Dev.

[29] Palma-Gudiel H, Córdova-Palomera A, Eixarch E (2015). Maternal psychosocial stress during pregnancy alters the epigenetic signature of the glucocorticoid receptor gene promoter in their offspring: a meta-analysis. Epigenetics.

[30] Radtke KM, Ruf M, Gunter HM (2011). Transgenerational impact of intimate partner violence on methylation in the promoter of the glucocorticoid receptor. Transl Psychiatry.

[31] Tobi EW, Slieker RC, Stein AD (2015). Early gestation as the critical time-window for changes in the prenatal environment to affect the adult human blood methylome. Int J Epidemiol.

[32] Mulligan CJ, D’Errico NC, Stees J (2012). Methylation changes at NR3C1 in newborns associate with maternal prenatal stress exposure and newborn birth weight. Epigenetics.

[33] Groenwold RHH, Moons KGM, Vandenbroucke JP (2014). Randomized trials with missing outcome data: how to analyze and what to report. CMAJ.

[34] Dumville JC, Torgerson DJ, Hewitt CE (2006). Reporting attrition in randomised controlled trials. BMJ.

[35] Houseman E, Accomando WP, Koestler DC (2012). DNA methylation arrays as surrogate measures of cell mixture distribution. BMC Bioinformatics.

[36] Langie SAS, Moisse M, Declerck K (2017). Salivary DNA Methylation profiling: aspects to consider for biomarker identification. Basic Clin Pharmacol Toxicol.

[37] van der Knaap LJ, Riese H, Hudziak JJ (2014). Glucocorticoid receptor gene (NR3C1) methylation following stressful events between birth and adolescence. The TRAILS study. Transl Psychiatry.

[38] Rubertsson C, Börjesson K, Berglund A (2011). The Swedish validation of Edinburgh Postnatal Depression Scale (EPDS) during pregnancy. Nord J Psychiatry.

[39] First MB, Spitzer RL, Gibbon M. Structured clinical interview for DSM-IV axis I disorders, patient edition (SCID-I/P, version 2.0). Washington: American Psychiatric Press; 1996.

[40] Beck AT, Ward CH, Mendelson M (1961). An inventory for measuring depression. Arch Gen Psychiatry.

[41] Beck AT, Epstein N, Brown G (1988). An inventory for measuring clinical anxiety: psychometric properties. J Consult Clin Psychol.

[42] Wechsler D. The Wechsler Preschool and Primary Scale of Intelligence, 3rd edition. San Antonio: TX Psychol Corp; 2002.

[43] Maksimovic J, Gordon L, Oshlack A (2012). SWAN: subset-quantile within array normalization for Illumina Infinium HumanMethylation450 BeadChips. Genome Biol.

[44] Pidsley R, Zotenko E, Peters TJ (2016). Critical evaluation of the Illumina MethylationEPIC BeadChip microarray for whole-genome DNA methylation profiling. Genome Biol.

[45] Benjamini Y, Hochberg Y (1995). Controlling the false discovery rate : a practical and powerful approach to multiple testing. J R Stat Soc B.

[46] Beck AT, Steer RA, Brown GK (1996). BDI-II manual.

[47] Beck AT, Steer RA (1990). Manual for the Beck anxiety inventory.

